# Identification of texture MRI brain abnormalities on first-episode psychosis and clinical high-risk subjects using explainable artificial intelligence

**DOI:** 10.1038/s41398-022-02242-z

**Published:** 2022-11-16

**Authors:** Alexandra I. Korda, Christina Andreou, Helena Victoria Rogg, Mihai Avram, Anne Ruef, Christos Davatzikos, Nikolaos Koutsouleris, Stefan Borgwardt

**Affiliations:** 1 Translational Psychiatry, Department of Psychiatry and Psychotherapy, University of Luebeck, Ratzeburger Allee 160, 23562 Lübeck, Germany; 2 Department of Psychiatry and Psychotherapy, Ludwig Maximilian University, Nussbaumstr. 7, 80336 Munich, Germany; 3 Department of Radiology, University of Pennsylvania School of Medicine, 3700 Hamilton Walk, Philadelphia, Pennsylvania 19104 USA

**Keywords:** Schizophrenia, Predictive markers

## Abstract

Structural MRI studies in first-episode psychosis and the clinical high-risk state have consistently shown volumetric abnormalities. Aim of the present study was to introduce radiomics texture features in identification of psychosis. Radiomics texture features describe the interrelationship between voxel intensities across multiple spatial scales capturing the hidden information of underlying disease dynamics in addition to volumetric changes. Structural MR images were acquired from 77 first-episode psychosis (FEP) patients, 58 clinical high-risk subjects with no later transition to psychosis (CHR_NT), 15 clinical high-risk subjects with later transition (CHR_T), and 44 healthy controls (HC). Radiomics texture features were extracted from non-segmented images, and two-classification schemas were performed for the identification of FEP vs. HC and FEP vs. CHR_NT. The group of CHR_T was used as external validation in both schemas. The classification of a subject’s clinical status was predicted by importing separately (a) the difference of entropy feature map and (b) the contrast feature map, resulting in classification balanced accuracy above 72% in both analyses. The proposed framework enhances the classification decision for FEP, CHR_NT, and HC subjects, verifies diagnosis-relevant features and may potentially contribute to identification of structural biomarkers for psychosis, beyond and above volumetric brain changes.

## Introduction

Structural brain abnormalities have been associated with schizophrenia and volume deficits progress across the trajectory of the illness [1–3]. These changes have been observed in early stages of psychosis as in first-episode psychosis patients (FEP) [4–6], have a wide distribution, affecting not only frontal, temporal, parietal cortical regions, but also subcortical, cerebellar, and callosal regions [7–9], and have a progressive course [10]. Several studies have identified similar volumetric disturbances in populations at high risk for psychosis [11, 12].

Psychosis risk definitions based on clinical criteria (clinical high risk, CHR) have low specificity for prediction of a future transition to overt psychosis, with only about a quarter of subjects developing a psychotic episode 3 years after diagnosis [13, 14]. Consequently, there has been major interest in brain structural changes as biomarkers useful for predicting the future emergence of psychosis in CHR with higher accuracy than clinical criteria alone. Several studies and meta-analyses investigated gray and white matter alterations in CHR have confirmed their potential as predictive indices, identifying a number of differences between CHR with later transition compared to those without in relation not only to total gray matter volume [15, 16], specific regions such as the anterior cingulate [17, 18], frontal cortex [19, 20], temporal cortex [21], parietal cortex [22], cerebellum [23], and insular cortex [24], but also white matter volume and structure [25–27].

The majority of the above studies have focused on regional volume or intensity measures and have not fully exploited the rich information contained in brain MRI, e.g., subtle differences between brain tissues or in the microstructure of the biological tissue [28], complex interrelations between different regions or gray, white matter and CSF [29]. Radiomics texture features are able to quantify the hidden patterns between voxel intensities and the spatial distribution of these patterns across brain regions. Meaningful comparison of texture feature results between different subjects is possible, when sMR images of the brain with similar resolution and noise levels are used, a common quantization method and the same number of gray levels in all quantized images [30, 31]. Radiomics texture features with their potential as image-based biomarkers have been widely used across several studies, like for cancer identification [32], Alzheimer’s [33] and Parkinson’s disease [34] as neurodegenerative diseases, major depression [35], and schizophrenia [36, 37]. In the field of schizophrenia research, texture features such as homogeneity and entropy have been shown to differentiate patients from healthy controls (HC) [38]. The main advantage of applying radiomics texture features is their potential to capture microscopic alterations in tissue characteristics of the brain [39], even though authors have stressed the significance of repeatability and reproducibility in applying texture radiomics features [40, 41].

In the current study, we applied radiomics texture features on FEP and CHR brain images for the first time. We examined potential differences between FEP, CHR with later transition to psychosis (CHR_T), CHR with no transition to psychosis (CHR_NT), and HC, by employing six texture feature maps extracted from non-segmented MR images and feeding into a deep neural network binary classification schema. Instead of applying conventional methods which show greater performance than deep neural networks [42], we employed an innovative approach that addresses a frequent concern about artificial intelligence methods, i.e., the explainability of results. Our goal is to gain insights into the examined disorders using radiomics texture features and explainable AI which achieve better performance in outcome modeling instead of statistical analysis of the radiomics texture features or the deep neural networks solely [31]. Our proposed algorithm integrates the complexity of the deep neural networks with the explainability of these networks as introduced by Bach et al. [43]. The algorithm, which is applied on non-segmented brain MRI for the investigation of the inter-relation between white matter (WM), GM and cerebrospinal fluid (CSF) has been previously reported for the identification of schizophrenia and major depression in Korda et al. [35]. Based on previous findings [44, 45], we hypothesize that radiomics texture feature models capture brain changes at microscale level which enable to (a) discriminate FEP patients from healthy controls and CHR subjects without a later transition to psychosis, and (b) predict later transition in CHR subjects based on the FEP pattern.

## Methods

### Study participants

The current analyses are based on data from the early detection of psychosis project (FePsy) at the Department of Psychiatry, University of Basel, Switzerland [46]. FePsy was a prospective clinical study of all consecutive referrals to the specialized early detection center of the two cantons Basel-Stadt and Basel-Landschaft (FePsy). FEP, CHR subjects, and healthy controls (HC) were recruited from November 2008 to April 2014. CHR subjects were followed up until transition (CHR-T) or, if no transition occurred (CHR-NT) for a maximum of 5 years. The Basel Screening Instrument for Psychosis (BSIP) was used for assessment of CHR and FEP status. The BSIP is a 46-item instrument based on variables that have been shown to be risk factors for early symptoms of psychosis such as DSM-III-R—“prodromal symptoms,” social decline, drug abuse, previous psychiatric disorders, or genetic liability for psychosis [47]. CHR status was defined either based on the presence of attenuated psychotic symptoms, brief limited intermittent psychotic symptoms, or having a first- or second-degree relative with a psychotic disorder and at least 2 additional risk factors for psychosis. FEP status and transition to psychosis were defined according to criteria by Yung et al. [48]: scores of 4 or higher on the BPRS’s hallucination item or scores of 5 on the BPRS’s strange thinking content, suspiciousness, or conceptual disarray items were necessary for inclusion. The symptoms must have lasted longer than a week and occured at least a couple of times a week. Patients with first-episode psychosis are those who on admission already fulfill the criteria for transition to psychosis as defined by the Yung et al. [48]. The Yung et al. (1998) criteria for the definition of psychosis represent a cutoff across the staging continuum and do not necessarily mean that these patients transitioned from a CHR state. CHR-T subjects were subjects classified as CHR at baseline (i.e., they had never achieved the psychosis cutoff according to Yung et al.), who transitioned to psychosis during follow-up. Thus, MRI was recorded at different stages across the psychosis continuum in CHR-T (before transition) and in FEP (after the emergence of overt psychosis). All participants for whom an MRI at baseline was available were included in the study.

Exclusion criteria for participants were age under 18 years old, a poor command of German, an IQ score below 70, prior psychotic episodes treated with antipsychotics for longer than 3 weeks, a clearly diagnosed brain disorder or substance dependency (other than cannabis dependence), or secondary psychotic symptoms within a depressive episode, bipolar disorder or borderline personality disorder. One of the 16 CHR-T participants took low-dose antipsychotic medication before having the MRI, the patient had received modest doses of atypical antipsychotic medication for behavioral control from the referring psychiatrist or general practitioner.

HC were recruited from the same geographical area as the CHR group through local advertisements and were matched to the CHR sample groupwise for age, gender, handedness, and education level. These individuals had no current psychiatric disorder, no history of psychiatric illness, head trauma, neurological illness, serious medical or surgical illness, substance dependency (except for cannabis and nicotine), and no family history of any psychiatric disorder as assessed by an experienced psychiatrist in a detailed clinical interview. The study was approved by the local ethics of northwestern and central Switzerland, and written informed consent was obtained from each participant. The study was conducted in accordance with the Declaration of Helsinki.

Structural MRI scans of 194 subjects were used for analysis: 77 FEP, 58 CHR_NT, 15 CHR_T, and 44 HC. Subjects were scanned using a SIEMENS (Erlangen, Germany) MAGNETOM VISION 1.5 T scanner at the University Hospital, Basel. A three-dimensional volumetric spoiled gradient recalled echo sequence generated 176 contiguous, 1 mm-thick sagittal slices. Imaging parameters were time-to-echo, 4 ms; time-to-repetition, 9.7 ms; flip angle, 12°; matrix size, 200 × 256; field of view, 25.6 × 25.6 cm matrix; voxel dimensions, 1.28 × 1 × 1 mm. Inclusion and exclusion criteria are described in detail in ref. [4].

### MRI data acquisition and data preprocessing

After inspection for artifacts and gross abnormalities, MRI scans were segmented into GM, WM, and CSF tissue maps in native space by means of the CAT12 toolbox (http://dbm.neuro.uni-jena.de), an extension of the SPM12 software package (Wellcome Department of Cognitive Neurology, London, England). All scans were reviewed by a neuroradiologist to rule out clinically significant abnormalities. The process was automated and has been described in Koutsouleris et al. [49] and Koutsouleris et al. [50]. Computation time of the preprocess was less than 30 min per subject.

It is critical to keep potential variances in image pose variance entering texture feature maps calculations to a minimum as these have been noted to affect texture estimates. In detail, the CAT12 toolbox extends the unified segmentation model consisting of MRI field intensity inhomogeneity correction, spatial normalization, and tissue segmentation in several preprocessing steps to further improve the quality of data preprocessing. Initially, the Optimized Blockwise Nonlocal-Means filter proposed by Coupe at al. [51] was applied to the MRI scans using the Rician noise adaption introduced in Wiest-Daesslé et al. [52] to increase the signal-to-noise ratio in the data. The usual strip artifacts in modulated images are greatly reduced by the default internal interpolation setting “Fixed 1 mm” in CAT12. Subsequently, an adaptive maximum a posteriori segmentation approach [53] extended by partial volume estimation [54] was employed to separate the MRI scans into GM, WM, CSF tissue. The segmentation step was finished by applying a spatial constraint to the segmented tissue probability maps based on a hidden Markov Random Field model [55] that removed isolated voxels which were unlikely to be a member of a certain tissue class and closed gaps in clusters of connected voxels of a certain class, resulting in a higher signal-to-noise ratio of the final tissue probability maps. The strength of the filters is automatically determined by estimating the residual noise in the image. The original voxels are projected into their new location in the warped images preserving the volume of a particular tissue within a voxel, i.e., produced by affine transformation (global scaling) and non-linear warping (local volume change), but this has the effect of introducing aliasing artifacts [56]. This latter effect was eliminated by applying discretization of the intensities in the mwp0* images (see section Adjust intensity values using histogram equalization), which is similar to applying a smoothing filter on the data distribution [57]. All scans were reviewed by a neuroradiologist to rule out clinically relevant abnormalities, data did not present any artifacts.

### Feature extraction

#### Adjust intensity values using histogram equalization

**Fig. 1:**
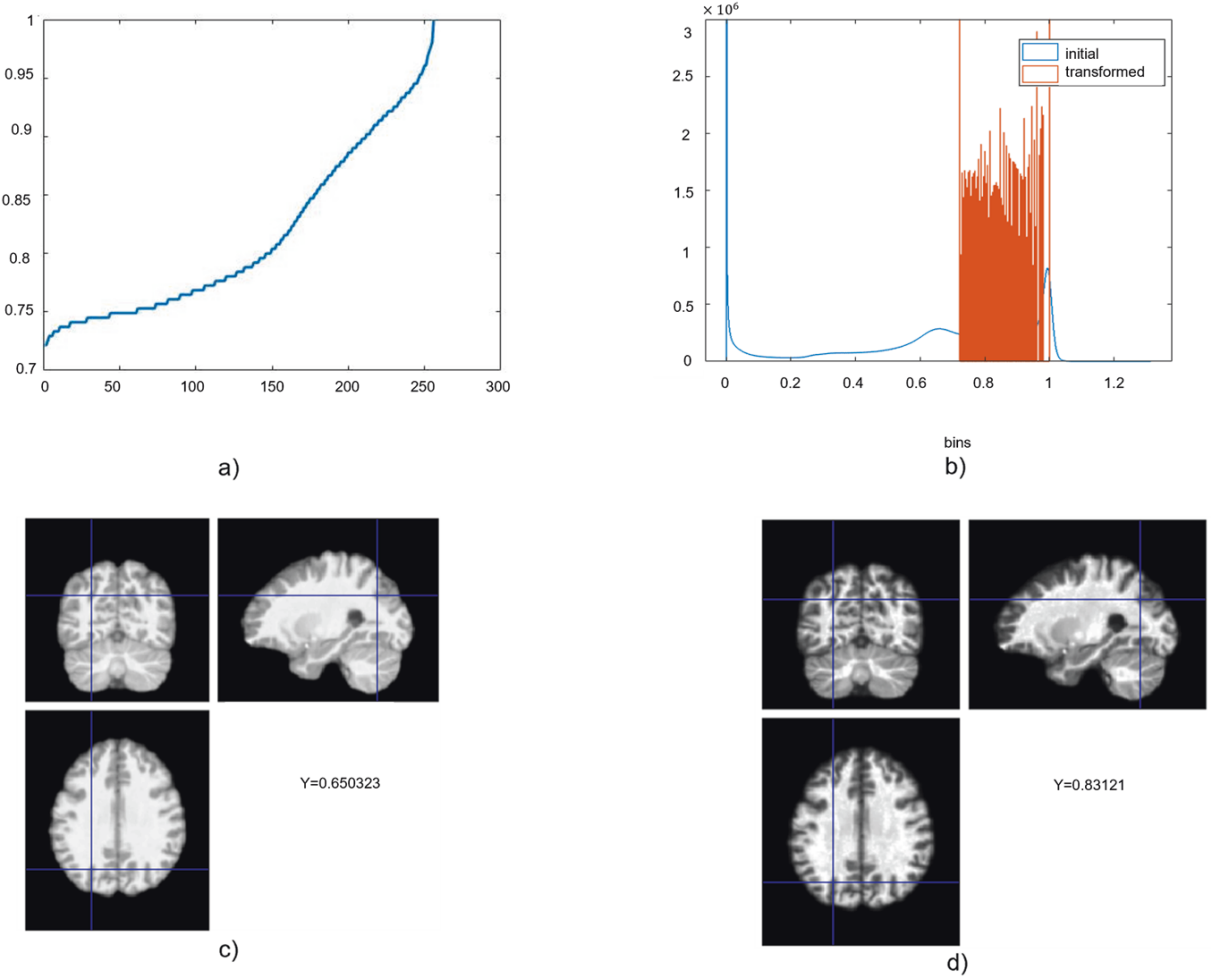
Histogram equalization to adjust the intensity values. Representation of **a** the discretization function and **b** the initial intensities and the adjusted intensities using histogram equalization. The brain MRI in SPM12 for the **c** initial MR image and **d** transformed MR image using the histogram equalization.

**Fig. 2:**
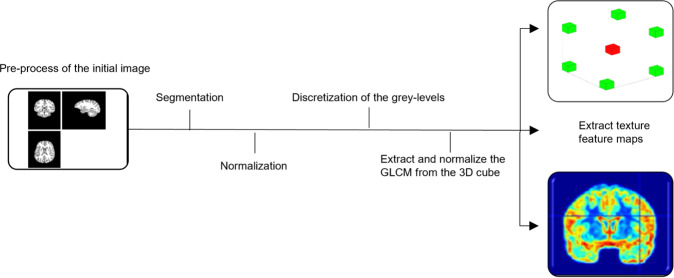
Workflow chart. Workflow for the calculation of the texture feature maps.

We used histogram equalization to adjust the contrast of a grayscale image. The original image has low contrast, with most pixel values in the middle of the intensity range. The *histeq* function in Matlab produces an output image with pixel values evenly distributed throughout the range and return a 1-by-256 vector that shows, for each possible input value, the resulting output value (see Fig. 1a). The number of bins normalizes images and forces the reproducibility of the texture features in new samples [57]. The brain sMRI used have similar resolution and noise levels, a common quantization method and the same number of gray levels in all quantized images was applied [58, 59]. In this study, we used the *histeq* function with a range of 2–256 bins (expresses the number of discrete gray levels), with a step of 2. The optimal number of the bins/bin-width (size) was selected in two stages. First, the images were inspected visually, and subsequently selected images were fed into the deep learning pipeline. Very large or small numbers of bins resulted in losing the brain boundaries between GM, WM, and CSF, while extremely noisy images returned. Finally, we exhaustively searched for the optimal number/width of bins by extracting the texture features across all images and feeding them one by one into the deep learning schema. The images with 16 bins returned the higher balanced accuracy. In Fig. 1, we have plotted the transformation curve for histogram equalization, the initial intensities, the adjusted intensities and the difference in the intensity between the initial and the transformed image is presented. The texture feature maps were extracted from the transformed mwp0* image (see Fig. 2 for workflow).

### Radiomics texture feature maps

We extracted textural parameters from non-segmented images using gray-level co-occurrence matrix (GLCM). Six texture features were calculated on the mwp0* images of the 194 subjects;1,164 individual feature maps in total. We extracted the texture features Entropy, Sum of Entropy, Difference of Entropy, Energy, Contrast and Homogeneity [60]. We a priori selected the examined features based on their connection to the morphological brain changes in FEP and CHR [30]. We selected the features that express opposite properties at microscale level (Entropy vs Energy and Homogeneity vs Contrast). Second order statistics of Entropy measure the arrangement of voxel gray-level intensities, depend on spatial relationship between gray-level intensities of the voxels [31], and have shown significant results in previous study [29]. The definitions of these features were as follows: GLCM-contrast reflects local variations in the GLCM; GLCM-energy reflects uniformity of gray-level voxel pairs; GLCM-entropy reflects randomness of gray-level voxel pairs; and GLCM-homogeneity reflects homogeneity of gray-level voxel pairs [61, 62]; finally GLCM-sum of entropy and difference of entropy reflect second order statistics of differentiation of gray-level distribution GLCM. We used voxel-by-voxel sliding 3D cube of 7 × 7 × 7 dimension as presented in a previous paper [35]. The GLCM matrix was normalized by dividing the values with the total sum of the values in the matrix. The normalization was performed for each GLCM extracted in each 3D cube independently. The texture features were extracted by a 3D 7 × 7 × 7 cube. Where the boundary of the cube touches non-zero brain gray levels, the algorithm maps the value to the center of the cube. For this reason, a 7 × 7 × 7 Gaussian kernel was used to smooth the voxel’s relevance for better localization of the results.

All the feature maps calculated from the 2d GLCM were basically a function of the probability of each GLCM entry and the difference of the gray levels, *g*_*1*_ and *g*_*2*_ [63]. We calculated feature maps only for cubes including non-zero values, as presented in Appendix A. The registered texture feature maps on the MNI space were fed one by one into a 10 × 10 nested cross-validation deep learning schema for group classification (see Supplemental Fig. 2).

We investigated the prediction accuracy of every single feature independently. This gives a physical meaning in the interpretation of the results. The first step was to select the features with the higher balanced accuracy and then to interpret the results in grouped fashion. We focus the description of results on the difference of entropy feature, which measures the randomness of intensity distribution in a second level, inherits the characteristics of entropy, and typically is less sensitive to outliers in a region [28]. The contrast gives a low weight to elements with similar gray-level values,but a high weight to elements with dissimilar gray levels, indicating large differences between neighboring voxels [58]. Texture feature map extraction from non-segmented brain images provides insight into voxel interrelationships of different modalities. To our knowledge, non-segmented images have never been used to detect the examined disorders due to the lack of interaction between these modalities as an indicator of diagnosis, suggesting a novel biomarker.

### Deep learning

The deep learning technique utilized the registered radiomics texture feature maps as input. We implemented two cycles of 10 times repeated nested cross-validation with 10 folds in the inner cycle and 10 folds in the outer cycle, resulting in 10,000 models. Feature selection (two-sample *t*-test) in the inner cycle, was cross-validated by selecting a number of features appropriate to the dimension of the database, namely, the top 200 ranked features that best discriminated the 2 classes in each classification schema (see section Classification results and visualization) [64]. The classifier implemented was a neural network-based classifier implemented in MATLAB (MathWorks Inc., Natick, Massachusetts, USA). The network used the hyperbolic tangent sigmoid transfer function and was batch-trained using the Levenberg-Marquardt training algorithm [65]. L2-regularization was applied to access possible types of uncertainty. We selected parameters after experimentation; 5 hidden layers (tested 2–5), each hidden layer consists of 2 nodes (tested 2–20) and 1000 epochs. The average balanced accuracy, sensitivity and specificity was calculated across all hold-out datasets of the 10 × 10 nested cross-validation, repeated 10 times.

### Visualization and evaluation of heatmaps

In order to perform localization, we calculated the relevance of the voxels in each class using the LRP algorithm for multilayer neural networks, as described in Bach et al. [43]. The explanation given by LRP would be a map showing which voxels of the original texture feature map contribute to the diagnosis and to what extent.

**Fig. 3:**
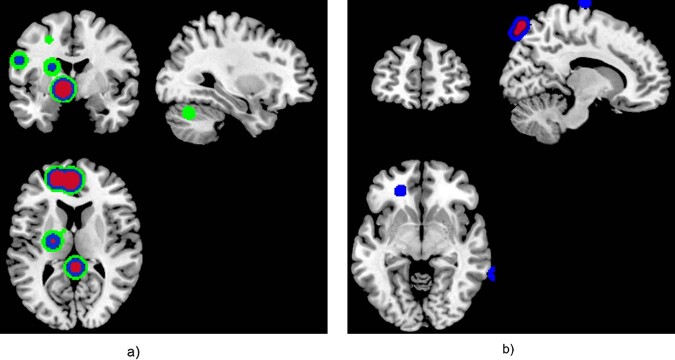
Visualization of the relevance of the voxels in each class for the classification schema (a). We demonstrated the smoothed PR with a 7 × 7 × 7 Gaussian kernel of the correct classified subjects of each group against the other in classification schema (a), FEP vs. HC for the registered texture feature map: **a** difference of entropy and **b** contrast. The red (cluster 1), blue (cluster 2), and green (cluster 3) color corresponds to the sorted clusters according to the number of subjects belong to each cluster.

**Fig. 4:**
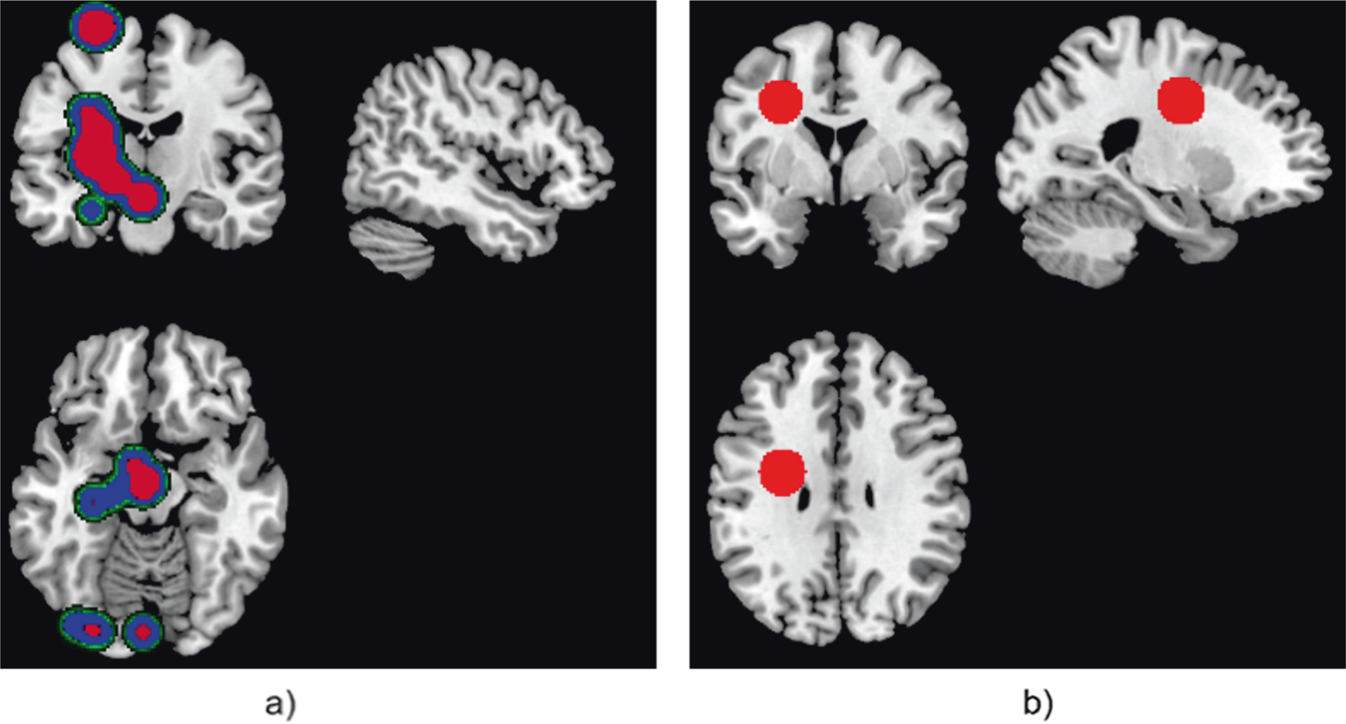
Visualization of the relevance of the voxels in each class for the classification schema (b). We demonstrated the smoothed PR with a 7 × 7 × 7 Gaussian kernel of the correct classified subjects of each group against the other in classification schema (b), FEP vs. CHR_NT for the registered texture feature map: **a** difference of entropy and **b** contrast. The red (cluster 1), blue (cluster 2), and green (cluster 3) color corresponds to the sorted clusters according to the number of subjects belong to each cluster.

**Fig. 5:**
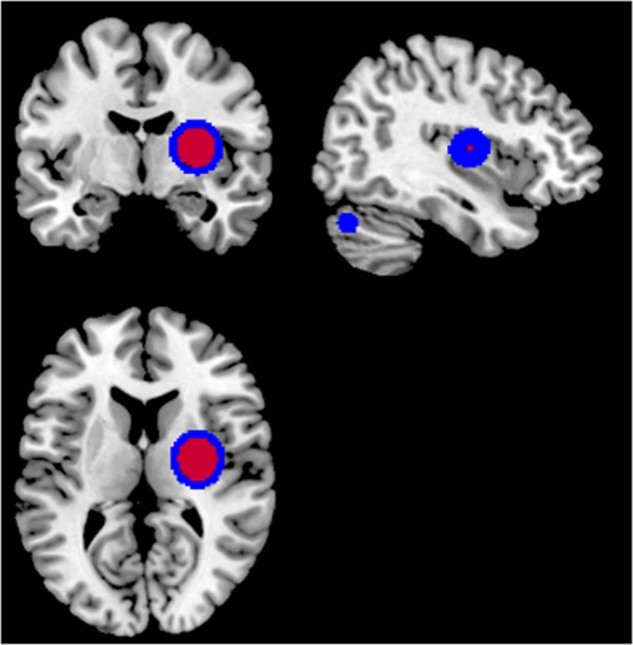
Visualization of the relevance of the voxels for CHR_T. Classification of CHR_T as FEP using the difference of entropy in classification schema (b). The red (cluster 1) and blue (cluster 2) color corresponds to the sorted clusters according to the number of subjects. The smoothed PR with a 7 × 7 × 7 Gaussian kernel is presented.

For the specific deep learning schema with 5 hidden layers and size 2, the calculation of the LRP algorithm is presented in Appendix B. The output of the LRP algorithm is a heatmap for each subject representing change in brain structures for FEP, CHR_NT, and CHR_T subjects. The final images were smoothed with a Gaussian filter of 10 mm and visualized using the MRIcron toolbox (https://people.cas.sc.edu/rorden/mricron/install.html). Similarities of the heatmaps for subjects in the same group were identified by clustering the heatmaps. Evaluation of the clustered heatmaps gives an estimation to the importance of each brain region to the diagnostic group membership (Figs. 3–5). Visualizations of the classification results on the hold-out dataset are presented in Figs. 3–5. The regions of interest were extracted using the AAL-VOIs atlas (https://neurovault.org/images/14257/). Additionally, we investigated cortical biomarkers for psychosis in GM, WM, and CSF. We used the JHU WM tractography atlas [66] to identify the WM tractography identified by the LRP.

### Clustering of subjects

Our intention was to display the heatmap of each correctly classified subject in the hold-out testing set produced by the LRP algorithm in a grouped fashion [67]. The affinity propagation (AP) algorithm [68] was selected to cluster the subject’s positive relevance, which uses the concept of message passing between the samples. The main advantage of the AP algorithm is that the number of clusters is not predefined. The input in the clustering algorithm is a matrix M × N, where N is the number of subjects and M is the relevance of each voxel. The output of the AP algorithm is a scalar for every subject that expresses in which cluster the subject belongs to. The average of the heatmaps from the subjects belonging to each cluster are represented.

For each classification process, the relevancies were classified using the AP algorithm. The clusters are presented in Figs. 3–5 for each texture feature map under the two-classification schema. The red color corresponds to the most intense cluster, the second one is in blue color and the green is the third one, where it exists (see Figs. 3–5).

## Results

### Sociodemographic characteristics

There were no significant differences between FEP and HC with respect to age and alcohol consumption. For the comparison between FEP and CHR, there were no significant differences with respect to age, years of education, smoking, alcohol consumption, and gender. There were significant differences between FEP and CHR with respect to BPRS total, BPRS Negative and Positives Symptoms, and SANS total (see Table 1).

**Table 1:** Group comparison was investigated using one-way ANOVA for continuous and χ^2^ test for categorical data.

One-way ANOVA (Welch’s)		
	F	*p*
FEP vs HC		
Age	1.63	0.204
Education (years)	19.26	<0.001
Smoking (cigarettes per day)	28.18	<0.001
FEP vs CHR		
Age	3.118	0.056
Education (years)	0.165	0.849
Global Assessment of Functioning (GAF)	46.875	<0.001
BPRS_Positive_Symptoms	19.498	<0.001
BPRS_Negative_Symptoms	22.494	<0.001
BPRS_total	462.930	<0.001
SANS_total	128.957	<0.001
Smoking (cigarettes per day)	2.418	0.102
χ² tests		
FEP vs HC		
Sex	13.6	<0.001
Alcohol	9.60	0.008
FEP vs CHR		
Sex	0.431	0.511
Alcohol	2.80	0.246

*BPRS* Brief Psychiatric Rating Scale, *GAF* Global Assessment of Functioning, *SANS* Scale for the Assessment of Negative Symptoms.

### Classification results and visualization

Two classifiers were developed in this study: (a) FEP vs. HC and (b) FEP vs. CHR_NT. The average balanced accuracy, sensitivity and specificity in the outer cycle were calculated for 10 folds in 10 repetitions. We tested the texture feature maps one-by-one in the deep learning classifier (see Tables 2–3). The specificity for FEP compared to HC (classifier a) was above 75% across features (Tables 2 and 3). The sensitivity in the classification schema (b) was above 70% across features, indicating adequately correct classification of FEP against CHR_NT.

**Table 2:** Presents the balanced accuracy, sensitivity, and specificity of the hold-out testing set, for the classification between FEP and HC.

Registered texture feature map	Balanced accuracy (%)	Sensitivity (%)	Specificity (%)
Entropy	66.55	56.93	75.08
Sum of entropy	72.02	68.22	**81.92**
Difference of entropy	**74.56**	69.58	**88.02**
Contrast	**75.24**	**76.48**	**83.63**
Homogeneity	70.43	63.87	77.23
Energy	70.29	60.71	**80.08**

The highest values are indicated in bold.

**Table 3:** Presents balanced accuracy, sensitivity, and specificity the hold-out testing set, for the classification between FEP and CHR_NT.

Registered texture feature map	Balanced accuracy (%)	Sensitivity (%)	Specificity (%)
Entropy	64.52	70.01	62.90
Sum of entropy	68.48	71.49	**73.20**
Difference of entropy	**72.01**	**79.29**	**71.00**
Contrast	**73.21**	**78.95**	**71.92**
Homogeneity	70.52	**78.59**	68.06
Energy	70.24	**75.59**	**72.30**

The highest values are indicated in bold.

In external validation using CHR-T, the most discriminative features were the difference of entropy and contrast resulting in a balanced accuracy above 72%; these features are presented in further detail for the interpretation of the results below; for completeness, analysis of the remaining texture features is presented in Supplement. Applying the classification schema (a), the feature difference of entropy classified 10 out of 15 CHR_T as FEP, while with the classification schema (b), the feature difference of entropy classified 11 out of 15 CHR_T as FEP patients.

In addition, both classification schemas were investigated using the non-segmented brain MR images, mwp0* images. The classification results were for the schema (a) 73.20% and (b) 68.98% balanced accuracy. The classification balanced accuracy is increased by applying the texture features and the explainability of the classification results are more informative. For comparison, voxel-based morphometry (VBM) analyses were performed in SPM12 toolbox on mwp1* images to identify volumetric brain differences between groups (a) FEP vs. HC, (b) CHR vs. HC and (c) FEP vs. CHR. There were no significant differences for corrected *p*-values (FWE < 0.05) in (a) and (b). Though, there were significant differences for corrected *p*-values (FWE < 0.05) in the between-group comparison of FEP vs. CHR in the left occipital lobe, see Supplemental Fig. 1. Demographic variables, Education (years), years of Smoking (Cigarettes per Day), alcohol, age, and sex were used as covariates. BPRS_total, BPRS_positive, BPRS_negative, GAF and SANS scores were used as additional covariates in comparison (b). There were no significant differences for corrected p-values (FWE < 0.05) in (a) and b) comparisons.

### Clustering of subjects

Across texture feature maps, regions with the highest contribution to the difference between FEP and HC (i.e., those with highest positive relevance PR) were in cerebellum and frontal gyrus (contrast), lenticular fasciclus (difference of entropy), see Fig. 3. In addition, regions most contributing to the difference between FEP and CHR_NT subjects were in parahippocampal, amygdala, precuneus, caudate, putamen, thalamus, hippocampus, insula, cerebellum, vermis, pallidum lingual, and motor area (difference of entropy and contrast), see Fig. 4. By explaining the classification decision using the LRP algorithm for the four models performed using the difference of entropy map and contrast map in classification schemas (a) and (b), the cerebellum was observed to be the key region for psychosis. Furthermore, key regions for psychosis were observed that are in line with previous studies, e.g., amygdala, caudate, insula, and hippocampus.

Regions contributing most to the CHR_T classification as FEP using the difference of entropy texture feature map (Fig. 5) were in parahippocampal areas, amygdala, precuneus, caudate, putamen, thalamus, hippocampus, insula, cerebellum, vermis, pallidum, lingual, and motor area. Furthermore, for the classification schema (a), two clusters of regions could be identified using the difference of entropy map. Cluster 1 included brain regions that were not part of the cluster 2 (Table 4), indicating a distinct neurobiological profile for subjects grouped in cluster 1.

**Table 4:** Presents the regions revealed in one of the cluster in FEP vs HC grouped fashion visualization.

FEP vs HC difference of entropy map	
Anterior corona radiata left	Cluster 1
Insular left	Cluster 1
Lateral fronto-orbital gyrus left	Cluster 1
Middle fronto-orbital gyrus left	Cluster 1
Gyrus rectus left	Cluster 1

We identified no consistent correlations of the PR with the volume of the regions indicated by the LRP when analyzed in the hold-out datasets that concluded to the highest balanced accuracy. The PR was negatively correlated with the volume of the regions indicated by the LRP for FEP subjects in classification schema (a) (Supplemental Fig. 5), such as optic tract left, corticospinal tract right, hypothalamus right (difference of entropy). Volumetric changes of regions such as calcarine left, fusiform right and cuneus left (contrast) were correlated positively with PR. In classification schema (b) (Supplemental Fig. 6), the PR for the FEP subjects was positively correlated with temporal and vermis (difference of entropy), frontal and precentral left (contrast) and uncorrelated with insula left, parahippocampal left, caudate left, calcarine left, Heschl left, cuneus left, putamen and thalamus left, lingual right (difference of entropy), frontal, temporal and parietal cortex, and supplementary motor area left (contrast). PR was uncorrelated with volumetric changes across CHR_NT that classified correctly against FEP such as thalamus, insula, putamen, hippocampus and parahippocampal, caudate, and amygdala (difference of entropy). CHR subjects who made a transition to psychosis and were classified as FEP with high balanced accuracy using the difference of entropy map in classification schema (b), presented uncorrelated volumetric changes with PR in many regions, i.e., thalamus, amygdala left, putamen, insula, and others shown in Fig. 7 in Supplement. These regions were the dominant regions for the prediction of psychosis based on baseline MRI.

For classification schema (a) (Fig. 3) LRP revealed PR in regions such as anterior corona radiata and genu of CC (contrast), posterior limb of internal capsule right, superior corona radiata right, superior fronto occipital fasciculus (difference of entropy). Regions such as cerebral peduncle right, posterior limb of internal capsule right and external capsule right (difference of entropy), body of CC, superior corona radiata right, and superior longitudinal fasciculus right (contrast) contribute more to the identification of the FEP subjects in classification schema (b) (Fig. 4) and the CHR_T subjects that classified as FEP subjects (Fig. 5). Across classification schemas, the WM volumetric changes did not impact the values of the PR indicating that texture features capture the dynamic inter-relation between GM, WM and CSF with predictive power for psychosis. Changes in the CSF captured by the difference of entropy for the FEP against CHR_NT (Fig. 4) and CHR_T classified as FEP (Fig. 5).

## Discussion

In this study, we used radiomic texture feature maps and the explainable AI method suggested by Bach et al. [43] to train and explain a classifier for psychosis. The model showed high balanced accuracy in classifying CHR subjects with a later transition as FEP rather than healthy subjects, thus indicating a potential use for predictive purposes. Importantly, texture features were not correlated with volumetric changes in a consistent manner, suggesting that this measure can reveal hidden neurobiological patterns expanding beyond volumetric changes of single regions to include the interrelations and borders between GM, WM, and CSF. Previous studies revealed subtle brain morphological changes in FEP and CHR subjects. Additionally, texture features measure at microscale level while brain regions are considered at macroscale level. Although, our understanding of the interactions between these various organizational scales is very limited [69].

Our findings are in line with a previous study applying texture analysis on MR data in patients with schizophrenia, which reported altered entropy in the hippocampus and the amygdala [38]. The regions contributing mostly to the decision of our classifier included key regions implicated in psychotic disorders in studies that have assessed gray and white matter changes in patients.

- Gray matter: Brain alterations with a decrease of intracranial and total brain volume have been reported in patients with chronic schizophrenia [70], particularly affecting cortical gray matter (and here predominantly in the prefrontal cortex (PFC)) [71]. Some of these changes such as volume decrease in the thalamus have been also observed in FEP [72], which emphasizes the presence of brain alterations already in early stages of the disease. These alterations might be pertinent to the transition to psychosis in CHR subjects: For example, CHR-T showed more deterioration over time in frontal and temporal regions than CHR_NT in early studies [73, 74]. In other regions such as the insular or cingulate cortex, gray matter loss in CHR-T has also been reported to exceed that of CHR_NT [75]. The structural alterations reported in previous studies are in line with the identified regions in the current analysis, such as temporal regions [76]; frontal cortex [77–79]; thalamus [72]; insula [80]; hippocampus and caudate nucleus [81, 82]; pallidum, putamen [83]; parahippocampus [83, 84]; and lingual cortex [85].
- White matter: There is accumulating evidence of compromised white matter function leading to abnormalities in synchronization and connectivity in patients with schizophrenia [86], the most widely used measure being fractional anisotropy (FA) assessed with diffusion tensor imaging (DTI). Neural changes, especially changes in the white matter connectivity could be observed throughout the different stages and progress of psychosis [87]. There are consistent findings of decreased FA, particularly in the inferior fronto-occipital fasciculus (IFOF) [88, 89]. Other studies have reported reduced white matter volume [90] and decreased FA in superior longitudinal fasciculus (SLF) [91] as well as in inferior fronto-orbital fasciculus (IFOF) [92–94], in CHR compared to FEP.

Multiple texture maps reveal significant contribution in diagnostic group membership for FA and IFOF, for both FEP and CHR_NT who classified as FEP. The above findings are in line with our results regarding the superior longitudinal fasciculus and inferior fronto-orbital fasciculus using contrast maps, and the thalamus, insula, hippocampus, pallidus, putamen, and parahippocampus using the difference of entropy maps. The contrast of the gray-level pairs reflects intracortical myelin as has been investigated for patients with schizophrenia in low-sensory and motor areas [95]. The difference of entropy in these regions potentially expresses the differentiation in the distribution of the pairwise gray-level randomness, which measures the brain subtle changes and express the entropy at microscale level. Entropy in psychosis has been investigated previously in patients with schizophrenia and indicated thalamus, hippocampus as potential brain biomarkers [29]. To exclude the possibility that our findings simply reflect volume reductions in these regions, we investigated whether regions contributing most to the diagnostic group membership (i.e., regions with PR values) were correlated with changes in brain volume in individuals with FEP and CHR (see Supplemental Figs 5–7). We found no indication of correlation of the relevance of the regions in the diagnostic group membership with volumetric changes in specific areas, i.e., amygdala, putamen, thalamus, hippocampus, insula, pallidus, rectus, parahippocampus, lingula, and Heschl.

The localization of alterations appeared to differ between subjects in different illness stages using the difference of entropy map: whereas most prominent alterations in FEP involved the cingulate gyrus and subcortical regions such as the nigrostriatal circuit, amygdala, and hypothalamus (Fig. 3a), CHR with later transition to psychosis demonstrated alterations mainly in cortical regions, the thalamus, and cerebellum (Fig. 4a). Both gray matter and white matter loss in specific brain regions were more prominent in CHR with transition to psychosis (CHR_T) compared to CHR who did make the transition. These brain alterations considered of high clinical relevance, resulting in—for example—more severe positive and negative symptoms and worse social functioning [91, 96].

The main advantages of the proposed method are the interpretability of the results and the use of non-segmented images, which eliminate segmentation errors. However, the implementation of the proposed method succumbs in limitations on the parameter’s selection. Many studies are showing significant differences in many texture features with variations in MRI acquisition [97–99]. The variability in standardization of MRI intensities influences the extraction of the texture features. In this study, the radiomic texture feature maps were extracted from the registered masked T1-weighted image in MNI space (as opposed to the original space as in Korda et al. [35]. The original intensities of T1-weighted MRI were not directly comparable across subjects, especially in the CHR and FEP subjects’s brain, so we proceeded to extract the GLCM texture features on the registered MRIs). Although there is great interest in using radiomics in health sciences, poor standardization and generalization of radiomics results hinder its application in clinical practice. Authors in [58], found that noise, resolution, choice of quantization method, and number of gray levels in the quantized image had a significant impact on most texture features, with the magnitude of the effect varying between features. Parameter selections such as the radius of the cubes and the structure of the neural network should be further examined. Regarding the LRP explanations, difference of entropy was the dominant texture feature map for psychosis. However, we recommend that further studies use multiple texture features, as each one expresses a different dynamic of brain heterogeneity. In summary, different methodological options need to be further explored in order to get a better understanding of the neurobiological changes in psychosis and their course from the CHR to the FEP stage, in order to make findings relevant for targeted interventions and individual treatment options. To investigate whether different neuroimaging modalities can be combined and used for increased accurate prediction, further work is required and should be addressed in future studies.

Another unresolved question concerns the effects of antipsychotic medication [3]. Several studies have shown that antipsychotic medication has an impact on alterations in WM disturbances between treated patients, drug-naïve patients and healthy controls [100, 101], while smaller brain tissue volumes and larger cerebrospinal fluid volumes can be observed in long-term treatment with antipsychotics [102]. It is still unclear whether brain abnormalities are already present at early stages and probably predicting the clinical onset of schizophrenia, or if these changes occur during the course of illness or are caused by pharmacological treatment [103]. It is crucial that the nature, time occurrence and further course of such brain changes as well as the impact of antipsychotic treatment is further investigated.

## Conclusions

Conventional imaging parameters are inadequate for quantification of the spatial distribution of microscopic tissue heterogeneity. A promising alternative to improve the diagnosis of psychosis on the basis of neurobiology is the application of radiomics texture features. We investigated the relation between neurobiological markers and LRP explanations. We observed that texture feature maps can be a useful representation for characterizing dissimilarities in brain structure in a complementary manner to volumetric analysis. Further studies in large cohorts are warranted to establish the key regions and key texture features that characterize psychotic disorders, in order to improve our understanding of the neurobiological changes that occur before the onset of psychosis and promote research on prevention and treatment methods for CHR subjects.

### Limitations

In this study, we investigated the prediction accuracy of different radiomics texture features separately. The number of features, especially when used for machine learning classification, is another challenging issue in radiomics’ utility. Further studies are needed to investigate the redundancy in the combination of the radiomics texture features. Although the results are in line with previous studies, an external validation set is required to validate the models and the potential biomarkers, which is not applied in this study. Repeated nested cross-validation and cross-validation feature selection is applied to avoid overfitting, minimize bias, and enhance the generalizability of the model. However, the small sample size is a drawback of the study and validation of the proposed method in larger datasets is requested. In contrast to conventional machine learning techniques, which fit better to small samples, deep learning techniques provide insights on the explanation for the classification decisions. Another methodological drawback of the proposed method lies on the missed consensus within texture features extraction process regarding the applied image normalization method. Last, in the current analysis the heterogeneity of diagnoses in the CHR_NT has not been investigated.

### Supplementary information

Supplemental material

## Data Availability

The datasets used and/or analyzed during the current study will be available from the authors upon reasonable request.

## Appendix A

Entropy: measure the complexity of the texture distribution. Entropy is a measure of chaos, if the values are consistently across, this means that the texture is very stochastic. Inverse to this property is the energy.

$${\mathrm{Entropy}} = - \mathop {\sum}\limits_{i = 1}^{N_{g_1}} {\mathop {\sum}\limits_{j = 1}^{N_{g_2}} {{\mathrm{GLCM}}(i,j)\ln {\mathrm{GLCM}}(i,j)} }$$

Contrast: reflects the distance from the GLCM diagonal. Values on the diagonal (where $i$ and $j$ are the same) result in zero contrast, whereas the contrast increases by increase of distance from the diagonal.

$${\mathrm{Contrast}} = \mathop {\sum}\limits_{i = 1}^{N_{g_1}} {\mathop {\sum}\limits_{j = 1}^{N_{g_2}} {(i - j)^2{\mathrm{GLCM}}(i,j)} }$$

Where *µ* is the GLCM mean.

Difference of entropy: measures the disorder related to the gray-level difference distribution of the image.

$${\mathrm{Diff}}_{{\mathrm{entropy}}} = - \mathop {\sum}\limits_{k = 0}^{N_g - 1} {{\mathrm{GLCM}}_{x - y}\left( k \right)\log _2({\mathrm{GLCM}}_{x - y}(k))}$$

where ${\mathrm{GLCM}}_{x - y}$, $N_g$ are expressed as:

$${\mathrm{GLCM}}_{x - y}(k) = \mathop {\sum}\limits_{i = 1}^{N_{g_1}} {\mathop {\sum}\limits_{j = 1}^{N_{g_2}} {{\mathrm{GLCM}}(i,j),{\mathrm{where}}\left| {i - j} \right| = k,{\mathrm{and}}\,k = 0,1,..,N_g - 1} }$$

Sum of entropy: measures the disorder related to the gray-level sum distribution of the image.

$${\mathrm{Sum}}_{{\mathrm{entropy}}} = - \mathop {\sum}\limits_{k = 2}^{2N_g} {{\mathrm{GLCM}}_{x + y}\left( k \right)\log _2({\mathrm{GLCM}}_{x + y}(k))}$$

$${\mathrm{GLCM}}_{x + y}\left( k \right) = \mathop {\sum}\limits_{i = 1}^{N_{g_1}} {\mathop {\sum}\limits_{j = 1}^{N_{g_2}} {{\mathrm{GLCM}}\left( {i,j} \right),{\mathrm{where}}\,i + j = k,{\mathrm{and}}\,k = 2,3,..,2N_g} }$$

Homogeneity: measures the smoothness (homogeneity) of the gray-level distribution of the image; it is (approximately) inversely correlated with contrast—if contrast is small, usually homogeneity is large

$${\mathrm{Homogeneity}} = \mathop {\sum}\limits_{i = 1}^{N_{g_1}} {\mathop {\sum}\limits_{j = 1}^{N_{g_2}} {\frac{1}{{1 + (i - j)^2}}{\mathrm{GLCM}}(i,j)} }$$

Energy: reflects the regularity and uniformity of the image distribution. The energy is high when the voxels are very similar, e.g., chess image.

$${\mathrm{Energy}} = \sqrt {\mathop {\sum}\limits_{i = 1}^{N_{g_1}} {\mathop {\sum}\limits_{j = 1}^{N_{g_2}} {{\mathrm{GLCM}}^2\left( {i,j} \right)} } }$$

## Appendix B

For the specific deep learning schema with 5 hidden layers with size 2, the LRP algorithm is presented:

Relevance of the 7th layer

$${{{\boldsymbol{R}}}}_{{{\boldsymbol{j}}}}^{\left( 7 \right)} = f\left( x \right),j = 1,2$$

Where the sixth layer is the real-valued prediction output of the classifier $f$ for the two classes $j$.

Relevance of the 6th layer between neurons i and j

For $$j = 1,2\,{{{\mathrm{and}}}}\,i = 1,2$$

$${{{\boldsymbol{R}}}}_{{{{\boldsymbol{i}}}} \leftarrow {{{\boldsymbol{j}}}}}^{\left( {6,7} \right)} = \left\{ {\begin{array}{*{20}{c}} {\frac{{z_{ij}}}{{z_j + \varepsilon }}R_j^{\left( 7 \right)},z_j \ge 0} \\ {\frac{{z_{ij}}}{{z_j - \varepsilon }}R_j^{\left( 7 \right)},z_j < 0} \end{array}} \right.,$$

$$z_{ij} = x_iw_{ij},$$

$${\boldsymbol{z}}_{\boldsymbol{j}} = \mathop {\sum}\limits_{i = 1}^{2} z_{ij} + b_j$$

Where $x_i$ is the output of the fifth hidden layer using the *tansig* transfer function on the net input, $w_{ij}$ are the weights and $b_j$ the biases of the neurons connect the fourth and third layer. $\varepsilon$ is *0.001* just to avoid the division with zero. So, the voxel-wise relevance in the third hidden layer is calculated as:

$${{{\boldsymbol{R}}}}_{{{\boldsymbol{i}}}}^{(6)} = \mathop {\sum}\limits_{j = 1}^2 {R_{i \leftarrow j}^{\left( {6,7} \right)}}$$

Relevance of the 5th layer between neurons i and k

For $$i = 1,2\,{{{\mathrm{and}}}}\,k = 1,2$$

$${{{\boldsymbol{R}}}}_{{{{\boldsymbol{k}}}} \leftarrow {{{\boldsymbol{i}}}}}^{\left( {5,6} \right)} = \left\{ {\begin{array}{*{20}{c}} {\frac{{z_{ki}}}{{z_i + \varepsilon }}R_i^{\left( 6 \right)}z_i \ge 0} \\ {\frac{{z_{ki}}}{{z_i - \varepsilon }}R_i^{\left( 6 \right)},z_i < 0} \end{array}} \right.,$$

$$z_{ki} = x_kw_{ki},$$

$${{{\boldsymbol{z}}}}_{{{\boldsymbol{i}}}} = \mathop {\sum}\limits_{k = 1}^2 {z_{ki} + b_i}$$

Where $x_k$ is the output of the fourth hidden layer using the *tansig* transfer function on the net input, $w_{ki}$ are the weights and $b_i$ the biases of the neurons connect the second and third layer. $\varepsilon$ is 0.001 just to avoid the division with zero. So, the voxel-wise relevance in the second hidden layer is calculated as:

$${{{\boldsymbol{R}}}}_{{{\boldsymbol{k}}}}^{\left( 5 \right)} = \mathop {\sum}\limits_{i = 1}^2 {R_{k \leftarrow i}^{\left( {5,6} \right)}}$$

Relevance of the 4th layer between neurons i and k

For $$i = 1,2\,{{{\mathrm{and}}}}\,k = 1,2$$

$${{{\boldsymbol{R}}}}_{{{{\boldsymbol{k}}}} \leftarrow {{{\boldsymbol{i}}}}}^{\left( {4,5} \right)} = \left\{ {\begin{array}{*{20}{c}} {\frac{{z_{ki}}}{{z_i + \varepsilon }}R_i^{\left( 5 \right)},z_i \ge 0} \\ {\frac{{z_{ki}}}{{z_i - \varepsilon }}R_i^{\left( 5 \right)},z_i < 0} \end{array}} \right.,$$

$$z_{ki} = x_kw_{ki},$$

$${{{\boldsymbol{z}}}}_{{{\boldsymbol{i}}}} = \mathop {\sum}\limits_{k = 1}^2 {z_{ki} + b_i}$$

Where $x_k$ is the output of the third hidden layer using the *tansig* transfer function on the net input, $w_{ki}$ are the weights and $b_i$ the biases of the neurons connect the second and third layer. $\varepsilon$ is 0.001 just to avoid the division with zero. So, the voxel-wise relevance in the second hidden layer is calculated as:

$${{{\boldsymbol{R}}}}_{{{\boldsymbol{k}}}}^{\left( 4 \right)} = \mathop {\sum}\limits_{i = 1}^2 {R_{k \leftarrow i}^{\left( {4,5} \right)}}$$

Relevance of the 3rd layer between neurons i and k

For $$i = 1,2\,{{{\mathrm{and}}}}\,k = 1,2$$

$${{{\boldsymbol{R}}}}_{{{{\boldsymbol{k}}}} \leftarrow {{{\boldsymbol{i}}}}}^{\left( {3,4} \right)} = \left\{ {\begin{array}{*{20}{c}} {\frac{{z_{ki}}}{{z_i + \varepsilon }}R_i^{\left( 4 \right)},z_i \ge 0} \\ {\frac{{z_{ki}}}{{z_i - \varepsilon }}R_i^{\left( 4 \right)},z_i < 0} \end{array}} \right.,$$

$$z_{ki} = x_kw_{ki},$$

$${{{\boldsymbol{z}}}}_{{{\boldsymbol{i}}}} = \mathop {\sum}\limits_{k = 1}^2 {z_{ki} + b_i}$$

Where $x_k$ is the output of the second hidden layer using the *tansig* transfer function on the net input, $w_{ki}$ are the weights and $b_i$ the biases of the neurons connect the second and third layer. $\varepsilon$ is 0.001 just to avoid the division with zero. So, the voxel-wise relevance in the second hidden layer is calculated as:

$${{{\boldsymbol{R}}}}_{{{\boldsymbol{k}}}}^{\left( 3 \right)} = \mathop {\sum}\limits_{i = 1}^2 {R_{k \leftarrow i}^{\left( {3,4} \right)}}$$

Relevance of the 2nd layer between neurons k and l

For $$k = 1,2\,{{{\mathrm{and}}}}\,l = 1,2$$

$${{{\boldsymbol{R}}}}_{{{{\boldsymbol{l}}}} \leftarrow {{{\boldsymbol{k}}}}}^{\left( {2,3} \right)} = \left\{ {\begin{array}{*{20}{c}} {\frac{{z_{lk}}}{{z_k + \varepsilon }}R_k^{\left( 3 \right)},z_k \ge 0} \\ {\frac{{z_{lk}}}{{z_k - \varepsilon }}R_k^{\left( 3 \right)},z_k < 0} \end{array}} \right.,$$

$$z_{lk} = x_lw_{lk},$$

$${{{\boldsymbol{z}}}}_{{{\boldsymbol{k}}}} = \mathop {\sum}\limits_{l = 1}^2 {z_{lk} + b_k}$$

Where $x_l$ is the output of the first hidden layer using the *tansig* transfer function on the net input, $w_{lk}$ are the weights and $b_k$ the biases of the neurons connect the second and third layer. $\varepsilon$ is 0.001 just to avoid the division with zero. So, the voxel-wise relevance in the first hidden layer is calculated as:

$${{{\boldsymbol{R}}}}_{{{\boldsymbol{l}}}}^{\left( 2 \right)} = \mathop {\sum}\limits_{k = 1}^2 {R_{l \leftarrow k}^{\left( {2,3} \right)}}$$

Relevance of the 1st layer between input voxels and neurons l

For $$d = 1, \ldots ,212295$$ voxels:

$${{{\boldsymbol{R}}}}_{{{{\boldsymbol{d}}}} \leftarrow {{{\boldsymbol{l}}}}}^{\left( {1,2} \right)} = \left\{ {\begin{array}{*{20}{c}} {\frac{{z_{dl}}}{{z_l + \varepsilon }}R_l^{\left( 2 \right)},z_l \ge 0} \\ {\frac{{z_{dl}}}{{z_l - \varepsilon }}R_l^{\left( 2 \right)},z_l < 0} \end{array}} \right.,$$

$$z_{dl} = x_dw_{dl},$$

$${{{\boldsymbol{z}}}}_{{{\boldsymbol{l}}}} = \mathop {\sum}\limits_{d = 1}^{212295} {z_{dl} + b_l}$$

Where $x_d$ is the input registered texture feature map based image, $w_{dl}$ are the weights and $b_l$ the biases of the neurons connect the input and second layer. So, the voxel-wise relevance in the input layer is calculated as:

$${{{\boldsymbol{R}}}}_{{{\boldsymbol{d}}}}^{\left( 1 \right)} = \mathop {\sum}\limits_{l = 1}^2 {R_{d \leftarrow l}^{\left( {1,2} \right)}}$$
