# Supplementary material for: Identification of texture MRI brain abnormalities on first-episode psychosis and clinical high-risk subjects using explainable artificial intelligence
Source: Transl Psychiatry. 2022 Nov 16;12:481. doi: 10.1038/s41398-022-02242-z (PMC9668814; doi:10.1038/s41398-022-02242-z)

**Supplement**

Visualization of between-group comparison results using VBM and the mwp1* images is presented in **Figure 1**. The registered texture feature maps on the MNI space were fed into a 10 times repeated 10x10 nested cross-validation deep learning scheme for group classification, see **Figure 2.**

Results of the rest four texture features examined are presented here:

*Grey matter:* Across texture feature maps, regions contributing mostly to the recognition of FEP compared to HC (**Figure 2**) were the thalamus, amygdala, striatum, hypothalamus, hippocampus, frontal gyrus, caudate nucleus, cerebellum (energy) and frontal gyrus (sum of entropy). Regions contributing mostly to the recognition of FEP patients against CHR_NT subjects (**Figure 3**) were parahippocampal, amygdala, precuneus, caudate, putamen, thalamus, hippocampus, insula, cerebellum, vermis, pallidum lingual and motor area (homogeneity). On the other hand, the region contributing mostly to the recognition of CHR_T subjects that classified in the FEP group (**Figure 4**) was located in putamen (energy).

*White matter:* For the classification schema a), regions that contribute to the recognition of FEP against HC were in fornix(cres)/stria terminalis, posterior thalamic radiation left, superior corona radiata right (energy), medial lemniscus left, posterior limb of internal capsule right, superior and posterior fronto-occipital fasciculus right, superior longitudinal fasciculus right, corona radiata right (entropy), splenium of corpus callosum and middle cerebellar peduncle (homogeneity), superior fronto-occipital fasciculus, middle cerebellar peduncle and posterior limb of internal capsule right (sum of entropy) (**Figure 2**). Regions that contribute to the recognition of FEP patients against CHR_NT were in cerebral peduncle right, posterior limb of internal capsule right and external capsule right, body of corpus callosum, superior corona radiata right and superior longitudinal fasciculus right, posterior corona radiata right (energy) (**Figure 3**). The same regions were contributed to the classification of CHR_T subjects as FEP using the energy map (**Figure 4**).

Across classification schemas, the white matter volumetric changes do not impact the values of the PR indicating that texture features capture interrelation voxels dynamic with predictive power for psychosis. Changes in the CSF captured by the entropy and sum of entropy maps for the classification schema a) (**Figure 2**) and the homogeneity map for the classification schema b) (**Figure 3**). Using the entropy, energy, homogeneity and sum of entropy maps no correlation was revealed between PR and grey matter volumetric changes across classification schemas.

**Figure 1:** Two sample t-test and corrected p-values (FWE<0.05) for FEP vs. CHR.

**
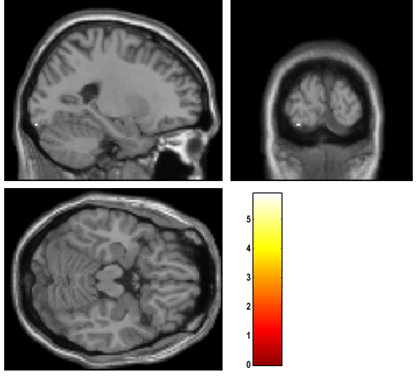
**

**Figure 2:** Nested cross-validation schema.

|  | Hold-out testing set |
| --- | --- |
|  | Training set splits into 10 folds |
|  | Validation set select the winner model |
|  | Training set |

10 folds

|  |  |  |  |  |  |  |  |  |  |  |  |  |  |  |  |  |  |  |  |  |
| --- | --- | --- | --- | --- | --- | --- | --- | --- | --- | --- | --- | --- | --- | --- | --- | --- | --- | --- | --- | --- |
|  |  |  |  |  |  |  |  |  |  |  |  |  |  |  |  |  |  |  |  |  |
|  |  |  |  |  |  |  |  |  |  |  |  |  |  |  |  |  |  |  |  |  |
|  |  |  |  |  |  |  |  |  |  |  |  |  |  |  |  |  |  |  |  |  |
|  |  |  |  |  |  |  |  |  |  |  |  |  |  |  |  |  |  |  |  |  |
|  |  |  |  |  |  |  |  |  |  |  |  |  |  |  |  |  |  |  |  |  |
|  |  |  |  |  |  |  |  |  |  |  |  |  |  |  |  |  |  |  |  |  |
|  |  |  |  |  |  |  |  |  |  |  |  |  |  |  |  |  |  |  |  |  |
|  |  |  |  |  |  |  |  |  |  |  |  |  |  |  |  |  |  |  |  |  |
|  |  |  |  |  |  |  |  |  |  |  |  |  |  |  |  |  |  |  |  |  |
|  |  |  |  |  |  |  |  |  |  |  |  |  |  |  |  |  |  |  |  |  |

Dataset

10 folds

10 repetitions

**Figure 3:** We demonstrated the smoothed PR with a 7x7x7 Gaussian kernel of the correct classified subjects of each group against the other in classification schema a), FEP vs. HC. The red (cluster 1), blue (cluster 2) and green (cluster 3) color corresponds to the sorted clusters according to the number of subjects belonging to each cluster.

| **Entropy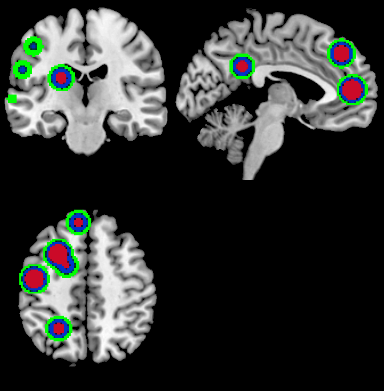** | **Sum of Entropy**  **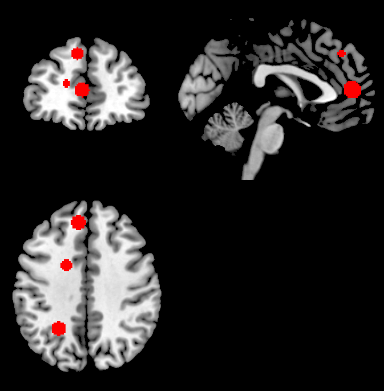** |
| --- | --- |
| **Homogeneity**  **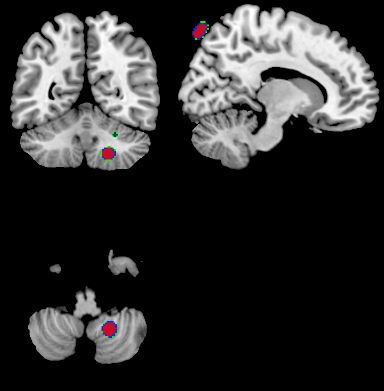** | **Energy**  **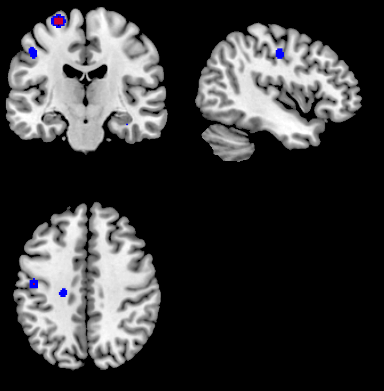** |
|  |  |

**Figure 4:** We demonstrated the smoothed PR with a 7x7x7 Gaussian kernel of the correct classified subjects of each group against the other in classification schema a), FEP vs. CHR_NT. The red (cluster 1), blue (cluster 2) and green (cluster 3) color corresponds to the sorted clusters according to the number of subjects belonging to each cluster.

| **Homogeneity**  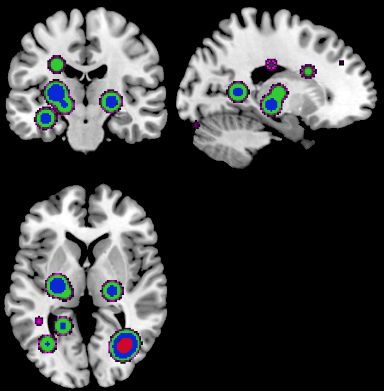 | **Energy**  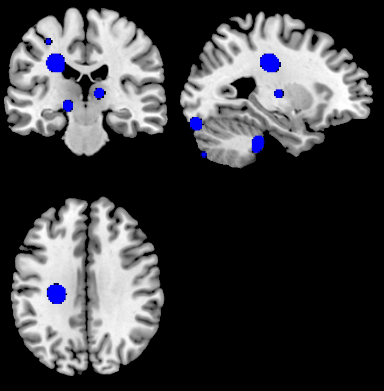 |
| --- | --- |

**Figure 5:** Classification of CHR_T as FEP using the Energy map.

**
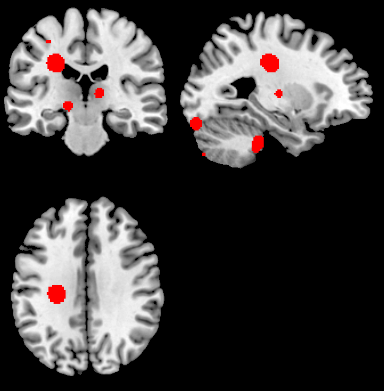
**

**Figure 6:** Correlation of the PR with the volumes for the correct classified FEP subjects in classification schema a) using the difference of entropy map a) and the contrast b).

**
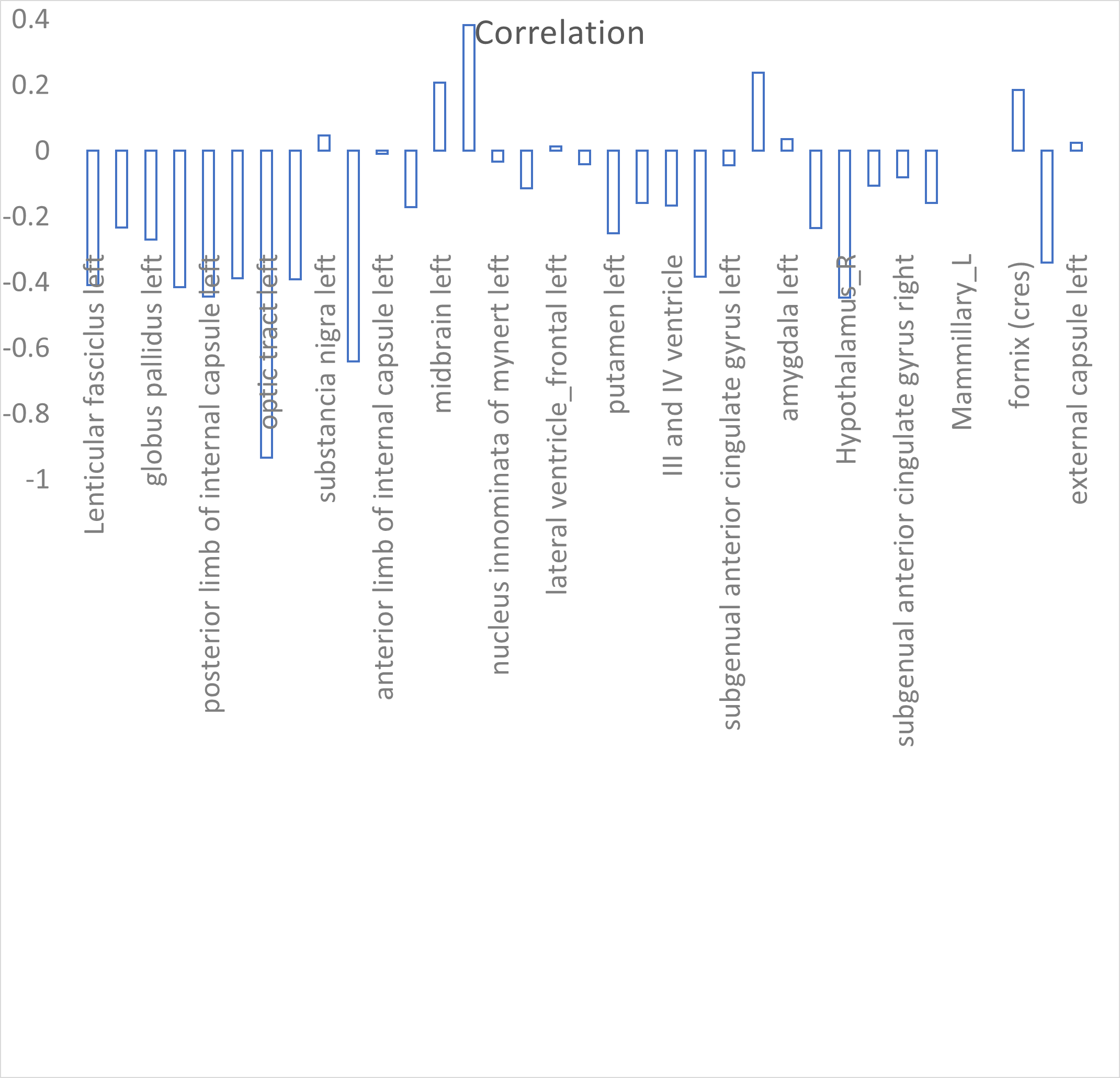
**

**a)**

**
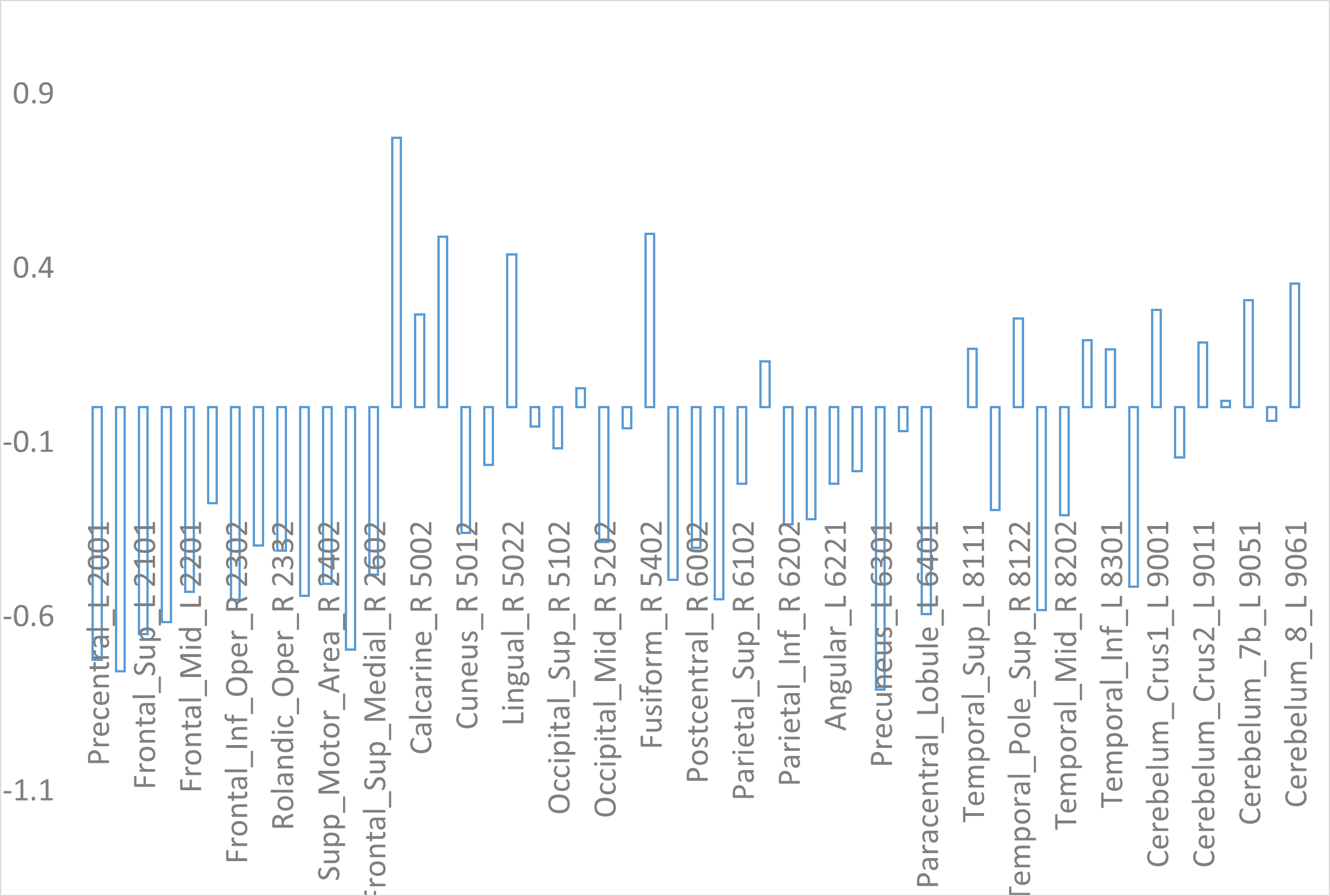
**

**b)**

**Figure 7:** Correlation of the PR with the volumes for the correct classified FEP subjects in classification schema b) using the difference of entropy map a) and the contrast b).


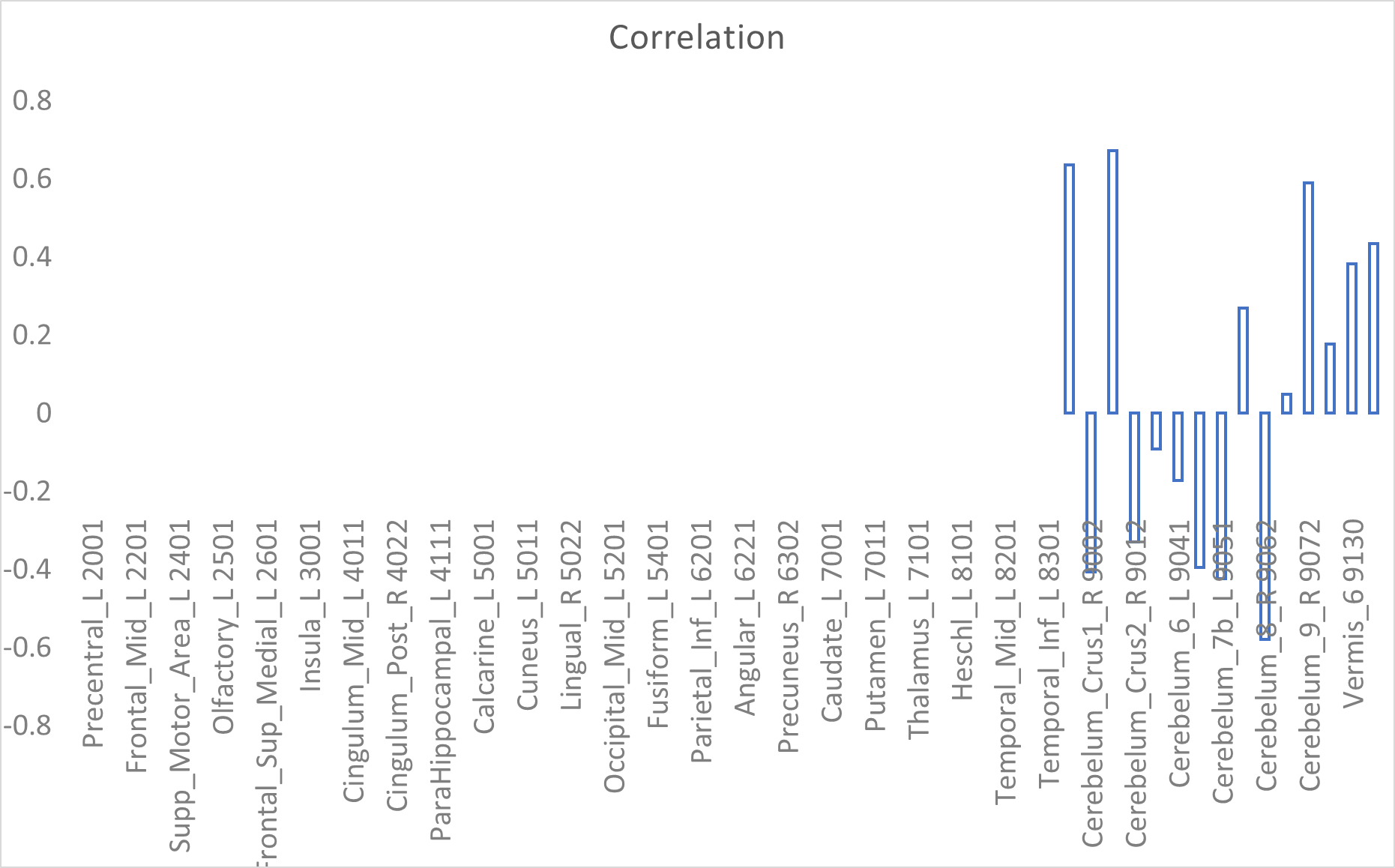


a)


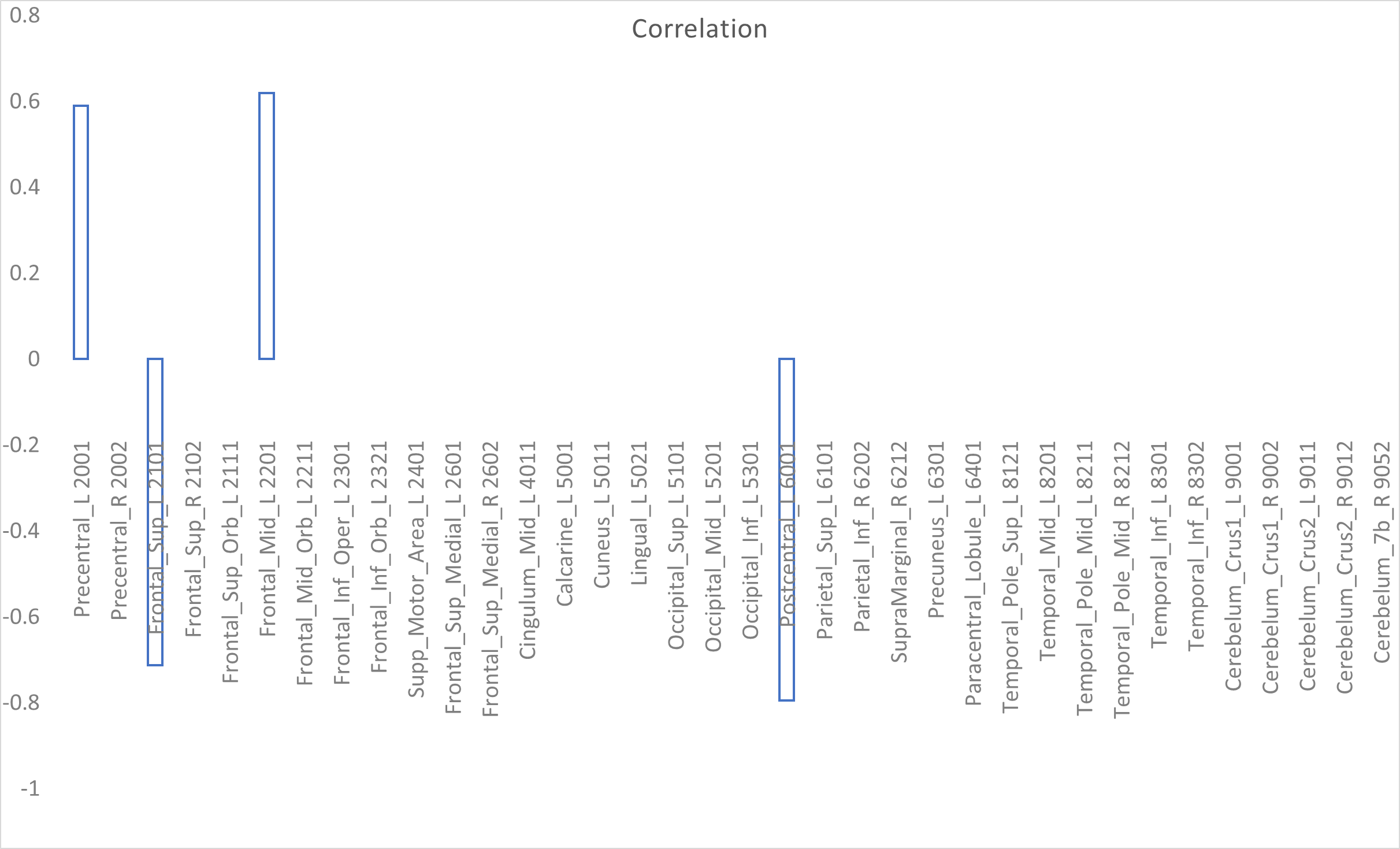


b)

**Figure 8:** Correlation of the PR with the volumes for the correct classified CHR_T subjects as FEP in classification schema b) using the difference of entropy map.


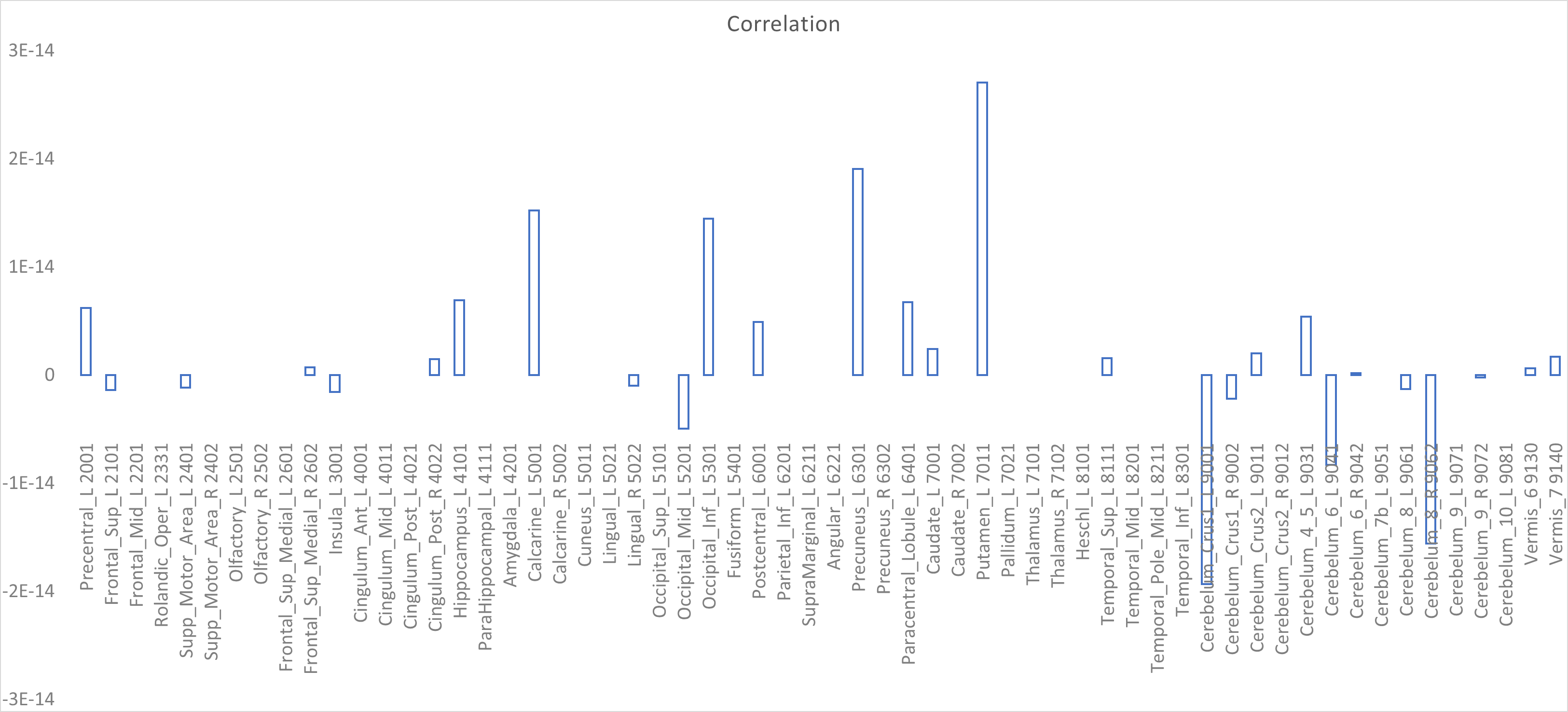

Supplement: Supplementary file 1 — Supplemental material [file 41398_2022_2242_MOESM1_ESM.docx]
